# Drug Repurposing in Dentistry: Towards Application of Small Molecules in Dentin Repair

**DOI:** 10.3390/ijms21176394

**Published:** 2020-09-02

**Authors:** Anahid A. Birjandi, Fernanda R. Suzano, Paul T. Sharpe

**Affiliations:** Centre for Craniofacial and Regenerative Biology, Guy’s Hospital, King’s College London, London SE1 9RT, UK; anahid.ahmadi_birjandi@kcl.ac.uk (A.A.B.); fernanda.suzano@kcl.ac.uk (F.R.S.)

**Keywords:** small molecules, Wnt pathway, GSK3, dentin repair, regeneration

## Abstract

One of the main goals of dentistry is the natural preservation of the tooth structure following damage. This is particularly implicated in deep dental cavities affecting dentin and pulp, where odontoblast survival is jeopardized. This activates pulp stem cells and differentiation of new odontoblast-like cells, accompanied by increased Wnt signaling. Our group has shown that delivery of small molecule inhibitors of GSK3 stimulates Wnt/β-catenin signaling in the tooth cavity with pulp exposure and results in effective promotion of dentin repair. Small molecules are a good therapeutic option due to their ability to pass across cell membranes and reach target. Here, we investigate a range of non-GSK3 target small molecules that are currently used for treatment of various medical conditions based on other kinase inhibitory properties. We analyzed the ability of these drugs to stimulate Wnt signaling activity by off-target inhibition of GSK3. Our results show that a c-Met inhibitor, has the ability to stimulate Wnt/β-catenin pathway in dental pulp cells in vitro at low concentrations. This work is an example of drug repurposing for dentistry and suggests a candidate drug to be tested in vivo for natural dentin repair. This approach bypasses the high level of economical and time investment that are usually required in novel drug discoveries.

## 1. Introduction

Small molecules are organic compounds of low molecular weight (<900 Daltons) that have the potential to regulate biological process. Owing to their small size, these molecules have the advantage of passing across cell membranes to reach targets, rendering them suitable for many targeted therapies. The attractive characteristics of small molecules makes them ideal for dental applications. However, discovery of novel drugs is typically associated with high risks and high levels of financial and time investment. In recent years, drug repurposing has been successful for identifying novel uses for approved or investigational drugs outside their original medical indication, contributing to substantial financial and time savings in preclinical and phase I and II stages [1]. Drug repurposing techniques comprise computation, experimental or mixed approaches. Systematic analysis of data such as gene expression and chemical structure are an example of computational approach. Experimental approaches on the other hand, comprise of molecular docking, signature matching, pathway mapping and genetic association [1]. Some examples of general drugs being repurposed include anticancer Zidovudine repurposed for HIV, anticancer Rituximab repurposed for rheumatoid arthritis, Thalidomide used for morning sickness repurposed for multiple myeloma, Raloxifene used for osteoporosis being repurposed for breast cancer [1]. An example of application of small molecules are those inhibiting the Wnt signaling pathway. Wnt signaling is an evolutionarily conserved pathway that regulates many crucial aspects of embryonic development and adult homeostasis such as cell fate determination, migration, polarity, neural patterning and organogenesis [2]. This pathway also regulates expression of many tumor-related proteins and typically shows abnormal activation in various cancer cells. Small molecules that block downstream stages of activation of this pathway and inhibit signal transduction can act as anticancer agents [3]. Furthermore, activation of Wnt/β-cat signaling has been demonstrated as an early response to damage in many tissues that can induce the repair process [2,4,5]. In this pathway, Axin2 is a negative regulator and a down-stream target. Glycogen synthase kinase 3 (GSK3) is a key enzyme of this pathway and a proline/serine protein kinase ubiquitously expressed and involved in many cellular signaling pathways controlling metabolism, differentiation, immunity, as well as cell death and survival [6]. In the presence of Wnt ligands, GSK-3 activity is inhibited, β-catenin can enter the nucleus to interact with Lef/Tcf transcription and express the target genes such as Axin2. In the absence of Wnt ligands, β-catenin and Axin2 are phosphorylated that leads to their ubiquitination and degradation [7]. GSK3 inhibitors have various forms and can have natural or synthetic sources that exhibit different mechanisms of action. They can be ATP-competitive inhibitors, non-ATP-competitive inhibitors, and substrate-competitive inhibitors [8]. Inhibition of GSK3 is a prime focus for targeting neurodegenerative and psychiatric disorders as well as behavioral impairments in Alzheimer’s disease [8,9]. Natural compounds, alkaloid Manzamine and the sesquiterpene Palinurin are non-ATP competitive GSK 3 inhibitors and have been shown to reduce tau phosphorylation in cell cultures [10,11]. Murine studies have shown an increased level of insulin growth factor in the brain after treatment with a GSK3 inhibitor Tideglusib (NP-12, NP03112) for five days. This growth factor exhibits therapeutic value for many neurodegenerative diseases [11,12,13]. GSK3 has also been identified as a pharmacological target for retinitis pigmentosa, an inherited condition leading to dystrophy and degeneration of retina and potential loss of vision [14].

In dentistry, one of the main goals is the maintenance of the natural structure of the tooth and provision of good and natural dental pulp protection when the tooth is damaged. While shallow tooth damage usually results in activation of odontoblast and formation of reactionary dentin that protects the pulp, deep cavities affect the pulp and jeopardize odontoblast survival. This results in activation of resident dental pulp stems, their proliferation and differentiation into new odontoblast-like cells and recruitment into the damage site to form reparative dentin [15,16]. This repair process is accompanied by rapid increase of Axin2 expression and differentiation of Axin2 expressing cells into odontoblast-like cells that will subsequently form reparative dentin [17]. Several GSK3 inhibitors have been shown to promote dentin repair in mice and rats with experimental pulp exposures [18,19]. Of these, Tideglusib is the only GSK3 drug that has to date been shown to be safe in patients [20,21,22]. In this study, we selected several small molecule drugs with anti-kinase activities that have been used in patients to treat several different conditions and investigated their potential to be repurposed for stimulation of reparative dentine. Table 1 lists the drugs used in this study, their mode of action and current applications. Separate in vitro studies have demonstrated these drugs to have hypoglycemic side effects and subsequent studies have shown their ability to inhibit GSK3 [23,24,25,26,27,28]. Therefore, we reasoned we could exhibit their potential to stimulate Wnt signaling pathway in the dental pulp cells. This approach enables us to identify potential candidate drug that can be ultimately used for dentin regeneration and help avoid the long financial and time investments for novel dental drug discoveries hence provide a faster route to clinical trials.

## 2. Results

### 2.1. Effective Concentrations and Cytotoxicity Testing

**Figure 1:**
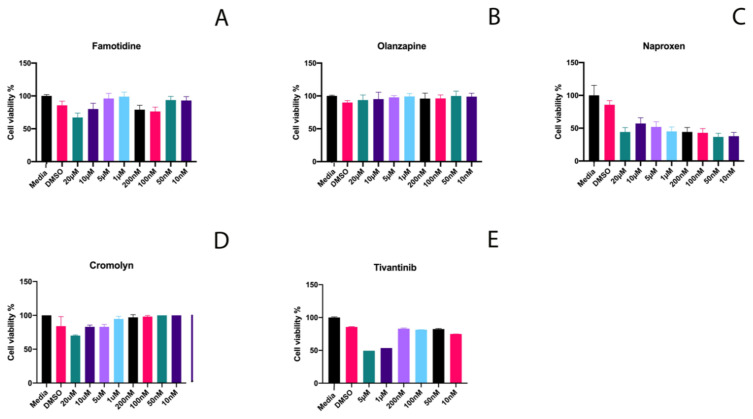
Viability of dental pulp cells at different concentrations of selected drugs. MTT cytotoxicity assay for (**A**) Famotidine, (**B**) Olanzapine, (**C**) Naproxen, (**D**) Cromolyn, and (**E**) Tivantinib.

To evaluate the potential of selected medications on the promotion of Wnt pathway activity and subsequently dentin/pulp damage repair, we first tested the effect of a range of concentrations of each drug on the viability of murine multipotent molar pulp cells. 17IA4 mouse dental pulp cells were incubated for 24 h with different concentrations of each drug. Cytotoxicity analyzed with the MTT assay determined the highest concentration of each drug that can be used in the subsequent stages (Figure 1). The highest concentrations for Famotidine and Olanzapine with the least effect on pulp cell viability were 5 µM and for Naproxen, 10 µM. These concentrations for Cromolyn and Tivantinib were 100 nM and 200 nM, respectively.

### 2.2. Potential to Promote Wnt Activity

**Figure 2:**
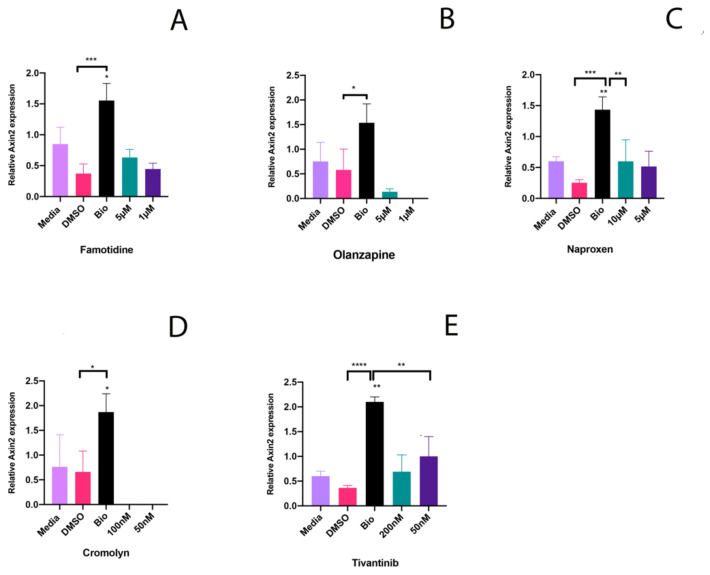
Ability of selected drugs to stimulate Wnt signaling in dental pulp. Axin2 qPCR for the highest non-toxic concentration of each drug with control (media only), DMSO and Bio 50 nM on 17IA4 cell line for (**A**) Famotidine * *p* = 0.0110, *** *p* = 0.0002, (**B**) Olanzapine * *p* = 0.0318, (**C**) Naproxen ** *p* = 0.0022 for Bio- Media and 0.0036 for Bio-10 µM, *** *p* = 0.0002, (**D**) Cromolyn * *p* = 0.0207 for Bio-DMSO and 0.034 for Bio-Media, and (**E**) Tivantinib ** *p* = 0.0018, **** *p* = <0.0001.

To test whether these drugs stimulate the Wnt signaling pathway, the two highest concentration of each drug that were non-toxic to pulp cells were assessed for their ability to induce Axin2 expression. For each drug, 17IA4 mouse dental pulp cells were incubated with selected concentrations in triplicate for 24 h. For controls, media, DMSO and Bio, a known Wnt agonist, were used. After 24 h, media and drug were removed, and cells were analyzed for expression of Axin2 by qPCR (Figure 2). Highest levels of Axin2 induction by Famotidine, Olanzapine and Naproxen were at 5 µM concentration. Cromolyn did not induce Axin2 expression at 100 nM and 50 nM. Tivantinib induced Axin2 expression at 200 nM and 50 nM with higher Axin2 induction at 50 nM.

### 2.3. Half Maximal Inhibitory Concentration of Tivantinib

**Figure 3:**
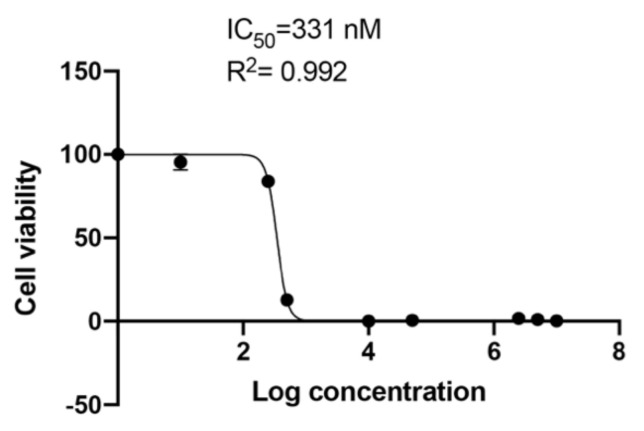
Determination of IC50 value of Tivantinib on 171A4 cells. Cells were seeded in 96-well plates and treated with Tivantinib at concentrations of 1 nM, 10 nM, 250 nM, 500 nM, 1 µM, 50 µM, 250 µM, 500 µM and 1 mM for 24 h. Following treatment, the percentage of cell survival was determined by MTT assay. The IC50 value of Tivantinib was determined by fitting a sigmoidal dose–response curve to the data, using the GraphPad Prism.

The expression of Axin2 in murine dental pulp cells at low concentrations of Tivantinib (50 nM) prompted us to select this drug for further analysis. To demonstrate the potency of Tivantinib, we measured its half maximal inhibitory concentration across a wide range of Tivantinib concentrations with a large scale MTT assay (Figure 3).

## 3. Discussion

Inhibition of GSK3 is paramount for targeting many conditions, such as neurodegenerative, psychiatric disorders, diabetes and cancer [8,9,39]. The potential application of drugs such as Famotidine and Naproxen in a dental context may seem unusual at first, but this study demonstrates how medications currently in the market can be repurposed for dental applications.

### 3.1. Kinase Inhibitory Properties of Investigated Drugs

Despite the different kinase inhibitory properties of the investigated drugs, they have all been shown to have the ability to also inhibit GSK3. This was demonstrated in separate invitro studies [23,24,25,26,27,28]. Famotidine is a competitive inhibitor of the histamine (H2) receptor, the dominant receptor involved in gastric acid secretion that abolishes the activation of adenylate cyclase induced by histamines. Famotidine is able to effectively suppress meal-stimulated acid secretion with higher efficiency than Ranitidine [29,30,31]. Olanzapine is an atypical antipsychotic agent used for treatment of schizophrenia. It is associated with side effects of metabolic abnormalities, weight gain and impaired glucose tolerance. This is suggested to be mainly through autophagy subsequent to the activation of AMP-activated protein kinase (AMPK) [23,32]. Through activation of autophagy through the AMPK pathway, Olanzapine is able to ameliorate the injury induced by Rotenone, a neurotoxic insecticide, in the PC12 cell line [40]. Famotidine and Olanzapine have been investigated for their potential to inhibit GSK3 due to their hypoglycemic side effects. Docking and experimental studies have demonstrated that Famotidine is fit within the binding pocket of GSK-3β and can decrease the glycemic response curve in animals [23,24,25].

Cromolyn is a mast cell stabilizer and a non-steroid antiasthma medication that blocks the release of inflammatory mediators [35,36]. It blocks the stimulation of alveolar macrophages by formyl peptide and leukotriene B4. It also blocks stimulation of phosphatidylinositol pathway [37]. Cromolyn inhibits the activity of protein kinase C (PKC). Regulation of mast cell secretory mechanism is thought involve PKC. Another suggested mechanism of action for Cromones is through a G-protein coupled receptor, GPR35, which modulates signaling via the Gi pathway [41,42,43]. Naproxen is a nonsteroidal anti-inflammatory drug (NSAIDS) that acts by inhibiting COX-1 and COX-2 and demonstrates antipyretic activities. Naproxen has been shown to target the phosphoinositide 3-kinase (PI3K) in urinary bladder cancer lines and rat bladder cancers [33]. NSAIDS also target MAP (ERK-2) kinase to inhibit the proliferation and growth of gastric cancer cell [34]. Both Cromolyn and Naproxen exhibit hypoglycemic properties and have been shown to inhibit GSK3 in vitro and in vivo. They were able to reduce the level of serum glucose and increase the level of hepatic glycogen and serum insulin in normal and diabetic mice [28]. Some of the anti-cancer properties of Naproxen and Cromolyn are attributed to their ability to inhibit GSK3 [44,45].

### 3.2. A c-Met Inibitor, Tivantinib, Induces Expression of Axin2 in Dental Pulp Cells at Low Concentration

Of the drugs studied here, Famotidine, Olanzapine and Naproxen did not affect viability of murine dental pulp cells at high concentrations. This was 5 µM for Famotidine and Olanzapine and 10 µM for Naproxen. Cromolyn and Tivantinib affected the viability of dental pulp cells at concentrations higher than 100 nM and 200 nM respectively. This suggests that these two drugs could potentially be candidates for in vivo trials if they also demonstrate the potential to promote the Wnt signaling pathway at such low concentrations. Cromolyn did not induce Axin2 expression and Famotidine, Olanzapine and Naproxen induced little Axin2 expression even at high concentrations. Tivantinib did however stimulate Axin2 expression at the low concentration of 50 nM although the level of Axin2 expression was lower than that observed with Bio (Figure 2). Bio is, however, a known Wnt agonist [46]. Therefore, the same level of Wnt stimulation may not be seen with other molecules such as Tivantinib.

Tivantinib is a non-ATP–competitive small molecule that selectively targets the c-Met receptor tyrosine kinase. Mass-spectrometry-based and chemical proteomics approaches contributed to the identification of GSK3 alpha and beta as novel tivantinib targets [26,27]. Tivantinib is a candidate anticancer agent for patients with hepatocellular carcinoma (HCC). A combination of Tivantinib, PI3K inhibitor LY294002 and mTOR inhibitor, rapamycin has been shown to largely inhibit the proliferation of glioblastoma cells [47]. Tivantinib was shown to inhibit GSK3α and to a lesser extent GSK3β in lung cancer cells [38]. Tivantinib was the first antiproliferative agent used in a phase III trials for Hepatocellular carcinoma. Although it was later announced that a phase III trial on Hepatocellular carcinoma did not improve overall survival, it was inferred that it is possible to perform integral tissue biomarker studies in patients with advanced hepatocellular carcinoma, pointing to the need for additional randomized studies to confirm whether MET inhibition can serve as a potential therapy for some patients with advanced hepatocellular carcinoma [48,49]. More than 500 kinases in the human kinome have conserved sites for ATP binding. Most kinase inhibitors are ATP-competitive and need to compete with ATP in their binding. The competition with high intracellular ATP levels can lead to discrepancy in measurements of cellular versus biochemical assays and demonstrate adverse secondary effects if used in chronic treatment. This is important when considering therapeutic approaches [8,26,50,51,52]. Non-ATP competitive inhibitors act by inducing a conformational shift in the target enzyme such that the kinase is no longer able to function [50]. They can therefore serve as better candidates due to enhanced potency and kinase selectivity. Tivantinib’s low IC50 demonstrates its potency and the non-ATP competitive nature of it is also an advantage (Figure 3).

Overall, our results show that a c-Met inhibitor used in a cancer trial had the lowest toxicity for dental pulp cells and the ability to stimulate Wnt/β-catenin pathway at low concentrations in vitro. We are currently investigating the in vivo effects of delivering Tivantinib into exposed pulp cavities via collagen sponges. The results presented here demonstrates an approach where drugs currently used in the medical field can be investigated and repurposed for potential dental application.

## 4. Materials and Methods

### 4.1. Cytotoxicity Assay

17IA4 cells were plated in 96-well plates at 20,000 cells/cm^2^ and incubated (37 °C, 5% CO_2_/95% air, 100% humidity) for 24 h using standard culture medium. Thereafter, the medium was replaced with conditioned (drugs + media) and control media (media alone) for another 24 h. To determine the cell metabolic activity, MTT (3-(4,5-Dimethylthiazol-2-yl)-2,5-diphenyltetrazolium bromide, Sigma Aldrich, St. Louis, MO, USA) was added to the controls and the conditioned media after 24 h. The resulting formazan product was then dissolved in 200 μL of dimethyl sulfoxide per well (DMSO, Sigma, St. Louis, MO, USA). A colorimetric plate reader (Thermo Multiskan Ascent 354 microplate reader) was used to read the absorbance at 540 nm with background subtraction at 630 nm.

### 4.2. Wnt Induction Assay

17IA4 cells were plated in 24-well plates and incubated (37 °C, 5% CO_2_/95% air, 100% humidity) for 24 h using standard culture medium. Selected drugs at the appropriate concentrations were used in triplicates to treat the cells for 24 h. Media only, DMSO and Bio (Sigma, St. Louis, MO, USA) were used as controls. Bio was used at 50 nM concentration. This is a safe concentration on pulp cell viability as mentioned before [18]. After 24 h, media was removed, and cells were lysed with Trizol for extraction of RNA and real time QPCR.

### 4.3. Real-Time qPCR Analysis

Total RNA was extracted from 17IA4 cells treated with different concentrations of selected drugs using Trizol (Life technologies, Carlsbad, CA, USA) as recommended by the manufacturer’s instructions. The RNA was reverse transcribed using random primers (M-MLV Reverse Transcriptase kit, Promega, Madison, WI, USA) according to the manufacturer’s instructions. Gene expression was then assayed by real-time qPCR using Syber Green (Roche, Basel, Switzerland) on a Rotor-Gene Q cycler (Qiagen, Hilden, Germany) system. Beta-actin was used as housekeeping gene (Forward-GGCTGTATTCCCCTCCATCG, Reverse-CCAGTTGGTAACAATGCCTGT) and Axin2 was the read-out for Wnt pathway activity (Forward-TGACTCTCCTTCCAGATCCCA, Reverse-TGCCCACACTAGGCTGACA. Reactions were performed in triplicate and relative changes to housekeeping gene were calculated by the 2^−ΔΔC T^ method where CT is the threshold cycle. Groups were then analyzed with one-way ANOVA followed up with multiple comparison tests in GraphPad Prism 8. Adjusted P value was reported.

### 4.4. IC50 Determination

171A4 Cells were seeded in 96-well plates and treated with 1 nM, 10 nM, 250 nM, 500 nM, 1 µM, 50 µM, 250 µM, 500 µM and 1 mM Tivantinib for 24 h in triplicate for each concentration. Following treatment, the percentage of cell survival was determined by MTT assay. The IC50 value of Tivantinib was determined by fitting a sigmoidal dose-response curve to the data, using the GraphPad Prism8. X values for concentration were transformed into scale. The Y values for MTT readouts were normalized to %. A linear regression analysis was performed using “dose response inhibition” and “log inhibitor versus normalized response”. The results were presented as IC50 and R square values.

## Figures and Tables

**Table 1:** List of drugs used in this study, their mode of action and current application.

Drug Name	Drug Type	Current Application	Reference
Famotidine	Histamine H2 blocker	Peptic ulcer	[29,30,31]
Olanzapine	Atypical antipsychotic	Bipolar disorders	[23,32]
Naproxen	Nonsteroidal anti-inflammatory drug	Pain relief	[33,34]
Cromolyn	Mast cell stabilizer	Mastocytosis	[35,36,37]
Tivantinib	c-Met Inhibitor	Hepatocellular carcinoma	[27,38]

## Abbreviations

GSK3	Glycogen synthase kinase 3
AMPK	AMP-activated protein kinase
PKC	Protein Kinase C
IC50	Half maximal inhibitory concentration

## References

[1] Pushpakom S., Iorio F., Eyers P.A., Escott K.J., Hopper S., Wells A., Doig A., Guilliams T., Latimer J., McNamee C. (2018). Drug repurposing: Progress, challenges and recommendations. Nat. Rev. Drug Discov..

[2] Fuerer C., Nusse R., ten Berge D. Wnt signalling in development and disease: Max Delbrück Center for molecular medicine meeting on Wnt signaling in development and disease. Proceedings of the EMBO Reports.

[3] Yan M., Li G., An J. (2017). Discovery of small molecule inhibitors of the Wnt/β-catenin signaling pathway by targeting β-catenin/Tcf4 interactions. Exp. Biol. Med..

[4] Whyte J.L., Smith A.A., Helms J.A. (2012). Wnt signaling and injury repair. Cold Spring Harb. Perspect. Biol..

[5] Minear S., Leucht P., Jiang J., Liu B., Zeng A., Fuerer C., Nusse R., Helms J.A. (2010). Wnt proteins promote bone regeneration. Sci. Transl. Med..

[6] Beurel E., Grieco S.F., Jope R.S. (2015). Glycogen synthase kinase-3 (GSK3): Regulation, actions, and diseases. Pharmacol. Ther..

[7] Clevers H., Nusse R. (2012). Wnt/β-Catenin Signaling and Disease. Cell.

[8] Eldar-Finkelman H., Martinez A. (2011). GSK-3 Inhibitors: Preclinical and Clinical Focus on CNS. Front. Mol. Neurosci..

[9] Hurtado D.E., Molina-Porcel L., Carroll J.C., Macdonald C., Aboagye A.K., Trojanowski J.Q., Lee V.M.Y. (2012). Selectively silencing GSK-3 isoforms reduces plaques and tangles in mouse models of Alzheimer’s disease. J. Neurosci..

[10] Hamann M., Alonso D., Martín-Aparicio E., Fuertes A., Pérez-Puerto M.J., Castro A., Morales S., Navarro M.L., del Monte-Millán M., Medina M. (2007). Glycogen Synthase Kinase-3 (GSK-3) Inhibitory Activity and Structure–Activity Relationship (SAR) Studies of the Manzamine Alkaloids. Potential for Alzheimer’s Disease. J. Nat. Prod..

[11] Martinez A., Gil C., Perez D.I. (2011). Glycogen Synthase Kinase 3 Inhibitors in the Next Horizon for Alzheimer’s Disease Treatment. Int. J. Alzheimers. Dis..

[12] Bolós M., Fernandez S., Torres-Aleman I. (2010). Oral Administration of a GSK3 Inhibitor Increases Brain Insulin-like Growth Factor I Levels. J. Biol. Chem..

[13] Torres-Aleman I. (2007). Targeting insulin-like growth factor-1 to treat Alzheimer’s disease. Expert Opin. Ther. Targets.

[14] Marchena M., Villarejo-Zori B., Zaldivar-Diez J., Palomo V., Gil C., Hernández-Sánchez C., Martínez A., de la Rosa E.J. (2017). Small molecules targeting glycogen synthase kinase 3 as potential drug candidates for the treatment of retinitis pigmentosa. J. Enzyme Inhib. Med. Chem..

[15] Couve E., Osorio R., Schmachtenberg O. (2014). Reactionary Dentinogenesis and Neuroimmune Response in Dental Caries. J. Dent. Res..

[16] Smith A.J., Cassidy N., Perry H., Bègue-Kirn C., Ruch J.V., Lesot H. (1995). Reactionary dentinogenesis. Int. J. Dev. Biol..

[17] Babb R., Chandrasekaran D., Carvalho Moreno Neves V., Sharpe P.T. (2017). Axin2-expressing cells differentiate into reparative odontoblasts via autocrine Wnt/β-catenin signaling in response to tooth damage. Sci. Rep..

[18] Neves V.C.M., Babb R., Chandrasekaran D., Sharpe P.T. (2017). Promotion of natural tooth repair by small molecule GSK3 antagonists. Sci. Rep..

[19] Zaugg L.K., Banu A., Walther A.R., Chandrasekaran D., Babb R.C., Salzlechner C., Hedegaard M.A.B., Gentleman E., Sharpe P.T. (2020). Translation Approach for Dentine Regeneration Using GSK-3 Antagonists. J. Dent. Res..

[20] Del Ser T., Steinwachs K.C., Gertz H.J., Andrés M.V., Gómez-Carrillo B., Medina M., Vericat J.A., Redondo P., Fleet D., León T. (2013). Treatment of Alzheimer’s disease with the GSK-3 inhibitor tideglusib: A pilot study. J. Alzheimers. Dis..

[21] Tolosa E., Litvan I., Höglinger G.U., Burn D., Lees A., Andrés M.V., Gómez-Carrillo B., León T., del Ser T. (2014). A phase 2 trial of the GSK-3 inhibitor tideglusib in progressive supranuclear palsy. Mov. Disord..

[22] Lovestone S., Boada M., Dubois B., Hüll M., Rinne J.O., Huppertz H.-J., Calero M., Andrés M.V., Gómez-Carrillo B., León T. (2015). A Phase II Trial of Tideglusib in Alzheimer’s Disease. J. Alzheimer’s Dis..

[23] Medak K.D., Townsend L.K., Hahn M.K., Wright D.C. (2019). Female mice are protected against acute olanzapine-induced hyperglycemia. Psychoneuroendocrinology.

[24] Mohammad M., Al-Masri I.M., Issa A., Al-Ghussein M.A.S., Fararjeh M., Alkhatib H., Taha M.O., Bustanji Y. (2013). Famotidine inhibits glycogen synthase kinase-3β: An investigation by docking simulation and experimental validation. J. Enzyme Inhib. Med. Chem..

[25] Mohammad M.K., Al-masri I.M., Taha M.O., Al-Ghussein M.A.S., AlKhatib H.S., Najjar S., Bustanji Y. (2008). Olanzapine inhibits glycogen synthase kinase-3β: An investigation by docking simulation and experimental validation. Eur. J. Pharmacol..

[26] Munshi N., Jeay S., Li Y., Chen C.R., France D.S., Ashwell M.A., Hill J., Moussa M.M., Leggett D.S., Li C.J. (2010). ARQ 197, a novel and selective inhibitor of the human c-Met receptor tyrosine kinase with antitumor activity. Mol. Cancer Ther..

[27] Remsing Rix L.L., Kuenzi B.M., Luo Y., Remily-Wood E., Kinose F., Wright G., Li J., Koomen J.M., Haura E.B., Lawrence H.R. (2014). GSK3 Alpha and Beta Are New Functionally Relevant Targets of Tivantinib in Lung Cancer Cells. ACS Chem. Biol..

[28] Motawi T.M.K., Bustanji Y., El-Maraghy S.A., Taha M.O., Al Ghussein M.A.S. (2013). Naproxen and cromolyn as new glycogen synthase kinase 3β inhibitors for amelioration of diabetes and obesity: An investigation by docking simulation and subsequent in vitro/in vivo biochemical evaluation. J. Biochem. Mol. Toxicol..

[29] Gwpach C., Fagot D., Emami S. (1989). Pharmacological control of the human gastric histamine H2 receptor by famotidine: Comparison with H1, H2 and H3 receptor agonists and antagonists. Eur. J. Clin. Investig..

[30] Smith L. (1985). Clinical Pharmacology of Famotidine. Digestion.

[31] (2012). Digestive System, Liver, and Abdominal Cavity. The Cat.

[32] Ikegami M., Ikeda H., Ishikawa Y., Ohsawa M., Ohashi T., Kai M., Kamei A., Kamei J. (2013). Olanzapine induces glucose intolerance through the activation of AMPK in the mouse hypothalamus. Eur. J. Pharmacol..

[33] Kim M.S., Kim J.E., Lim D.Y., Huang Z., Chen H., Langfald A., Lubet R.A., Grubbs C.J., Dong Z., Bode A.M. (2014). Naproxen induces cell-cycle arrest and apoptosis in human urinary bladder cancer cell lines and chemically induced cancers by targeting PI3K. Cancer Prev. Res..

[34] Husain S.S., Szabo I.L., Pai R., Soreghan B.A., Tarnawski A.S. (2001). MAP (ERK-2) kinase—A key target for NSAID-induced inhibition of gastric cancer cell proliferation and growth. Gastroenterology.

[35] Murphy S., Kelly H.W. (1987). Cromolyn Sodium: A Review of Mechanisms and Clinical Use in Asthma. Drug Intell. Clin. Pharm..

[36] Storms W., Kaliner M.A. (2005). Cromolyn sodium: Fitting an old friend into current asthma treatment. J. Asthma.

[37] Holian A., Hamilton R., Scheule R.K. (1991). Mechanistic aspects of cromolyn sodium action on the alveolar macrophage: Inhibition of stimulation by soluble agonists. Agents Actions.

[38] Kuenzi B.M., Remsing Rix L.L., Kinose F., Kroeger J.L., Lancet J.E., Padron E., Rix U. (2019). Off-target based drug repurposing opportunities for tivantinib in acute myeloid leukemia. Sci. Rep..

[39] Maqbool M., Hoda N. (2017). GSK3 Inhibitors in the Therapeutic Development of Diabetes, Cancer and Neurodegeneration: Past, Present and Future. Curr. Pharm. Des..

[40] Xiong Y., Song Y., Zhu Y., Zuo W., Zhao Y., Shen X., Wang W., Liu Y., Wu J., Liang Z. (2020). Neuroprotective effects of olanzapine against rotenone-induced toxicity in PC12 cells. Acta Pharmacol. Sin..

[41] Wang L., Correia I., Basu S., Theoharides T.C. (1999). Ca2+ and phorbol ester effect on the mast cell phosphoprotein induced by cromolyn. Eur. J. Pharmacol..

[42] Lucas A.M., Shuster S. (1987). Cromolyn inhibition of protein kinase C activity. Biochem. Pharmacol..

[43] Wang J., Simonavicius N., Wu X., Swaminath G., Reagan J., Tian H., Ling L. (2006). Kynurenic Acid as a Ligand for Orphan G Protein-coupled Receptor GPR35. J. Biol. Chem..

[44] Motawi T.M.K., Bustanji Y., El-Maraghy S., Taha M.O., Al-Ghussein M.A.S. (2014). Evaluation of naproxen and cromolyn activities against cancer cells viability, proliferation, apoptosis, p53 and gene expression of survivin and caspase-3. J. Enzyme Inhib. Med. Chem..

[45] Li R., Ou J., Li L., Yang Y., Zhao J., Wu R. (2018). The Wnt signaling pathway effector TCF7L2 mediates olanzapine-induced weight gain and insulin resistance. Front. Pharmacol..

[46] Sato N., Meijer L., Skaltsounis L., Greengard P., Brivanlou A.H. (2004). Maintenance of pluripotency in human and mouse embryonic stem cells through activation of Wnt signaling by a pharmacological GSK-3-specific inhibitor. Nat. Med..

[47] Wu Y., Li Z., Zhang L., Liu G. (2019). Tivantinib hampers the proliferation of glioblastoma cells via PI3K/Akt/Mammalian Target of Rapamycin (mTOR) signaling. Med. Sci. Monit..

[48] Best J., Schotten C., Lohmann G., Gerken G., Dechêne A. (2017). Tivantinib for the treatment of hepatocellular carcinoma. Expert Opin. Pharmacother..

[49] Rimassa L., Assenat E., Peck-Radosavljevic M., Pracht M., Zagonel V., Mathurin P., Rota Caremoli E., Porta C., Daniele B., Bolondi L. (2018). Tivantinib for second-line treatment of MET-high, advanced hepatocellular carcinoma (METIV-HCC): A final analysis of a phase 3, randomised, placebo-controlled study. Lancet Oncol..

[50] Garuti L., Roberti M., Bottegoni G. (2010). Non-ATP Competitive Protein Kinase Inhibitors. Curr. Med. Chem..

[51] Bamborough P., Drewry D., Harper G., Smith G.K., Schneider K. (2008). Assessment of chemical coverage of kinome space and its implications for kinase drug discovery. J. Med. Chem..

[52] Martinez A., Castro A., Dorronsoro I., Alonso M. (2002). Glycogen synthase kinase 3 (GSK-3) inhibitors as new promising drugs for diabetes, neurodegeneration, cancer, and inflammation. Med. Res. Rev..

